# A recurrent deletion on chromosome 2q13 is associated with developmental delay and mild facial dysmorphisms

**DOI:** 10.1186/s13039-015-0157-0

**Published:** 2015-07-31

**Authors:** Eva Hladilkova, Tuva Barøy, Madeleine Fannemel, Vladimira Vallova, Doriana Misceo, Vesna Bryn, Iva Slamova, Sarka Prasilova, Petr Kuglik, Eirik Frengen

**Affiliations:** Department of Medical Genetics, University of Oslo and Oslo University Hospital, P.O.Box 1036, Blindern, N-0315 Oslo, Norway; Department of Medical Genetics, University Hospital, Children Medical Hospital, Brno, Czech Republic; Department of Genetics and Molecular Biology, Institute of Experimental Biology, Faculty of Science, Masaryk University, Kamenice 5, 625 00 Brno - Bohunice, Czech Republic; Department of Habilitation, Sykehuset Innlandet HF, Lillehammer, Norway; Sanatorium Helios ltd., Laboratory of Medical Genetics, Brno, Czech Republic

**Keywords:** 2q13 deletion, Developmental delay, Facial dysmorphism, aCGH, *BCL2L11*, *FBLN7*, *TMEM87B*

## Abstract

We report two unrelated patients with overlapping chromosome 2q13 deletions (patient 1 in chr2:111415137-113194067 bp and patient 2 in chr2:110980342-113007823 bp, hg 19). Patient 1 presents with developmental delay, microcephaly and mild dysmorphic facial features, and patient 2 with autism spectrum disorder, borderline cognitive abilities, deficits in attention and executive functions and mild dysmorphic facial features. The mother and maternal grandmother of patient 1 were healthy carriers of the deletion. Previously, 2q13 deletions were reported in 27 patients, and the interpretation of its clinical significance varied. Our findings support that the 2q13 deletion is associated with a developmental delay syndrome manifesting with variable expressivity and reduced penetrance which poses a challenge for genetic counselling as well as the clinical recognition of 2q13 deletion patients.

**Electronic supplementary material:**

The online version of this article (doi:10.1186/s13039-015-0157-0) contains supplementary material, which is available to authorized users.

## Background

Genome-wide analyses performed on large numbers of patients have led to the discovery of a multitude of copy-number variations (CNVs). Segmental duplications predispose genomic regions to recurrent duplication and deletion by non-allelic homologous recombination (NAHR) events, some of which cause clinically recognizable core phenotypes, e.g., Angelman, Prader-Willi, Smith-Magenis and Williams-Beuren syndromes (reviewed by [1]). Other imbalances, such as deletions in chromosome 1q21, 15q11, 15q13 and 16p11, present with significant clinical variability [2]. For example the chromosome 1q21.1 deletion is found in patients manifesting one of several features including intellectual disability (ID), autism spectrum disorders (ASDs), schizophrenia, microcephaly, cataracts and congenital heart defects, and it is also detected in healthy carriers [3, 4]. Similarly the chromosome 16p11.2 deletion is detected in patients presenting with a variable combination of clinical features including ID, ASDs, cardiac defects, speech delay, obesity and dysmorphic features, as well as in healthy carriers [5–7].

Recurrent genomic imbalances involving the chromosome 2q13 (chr2:110-114 Mb) are less described. So far 27 patients carrying a 2q13 deletion have been reported [8–15]. The patients present with an apparently unspecific and variable clinical phenotype, including developmental delay (DD), ASDs, attention deficits hyperactivity disorder (ADHD), heart defects and craniofacial abnormalities, and several healthy carriers have been identified [8, 9, 11, 15]. Still, the clinical significance of the 2q13 deletion is not fully determined.

We describe two additional, unrelated patients carrying a deletion in 2q13. Patient 1 presents with DD, microcephaly and mild dysmorphic features, and patient 2 with ASD, borderline cognitive ability, attention and executive function deficits and mild dysmorphic features. The reduced penetrance associated with this deletion syndrome is supported by the identification of two generations of healthy carriers in one of the families.

## Case presentation

**Fig. 1:**
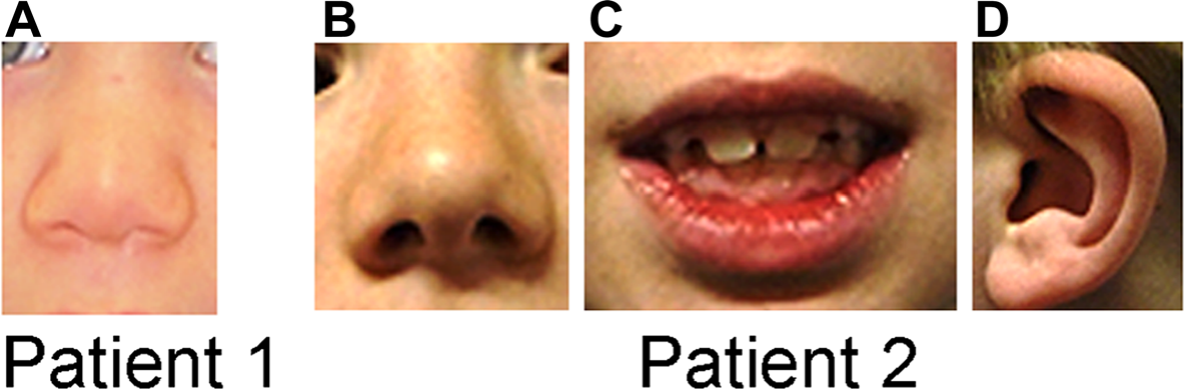
Patient 1: detail of nose (**a**) and patient 2: detail of nose (**b**), mouth (**c**) and ear (**d**). (description in the text)

Patient 1, a girl, was the first child born to non-consanguineous healthy Czech parents. The birth was induced at gestational week 41 and the amniotic fluid was highly turbid. Birth weight was 3500 g (75^th^ centile), length 50 cm (50^th^ centile), and occipitofrontal circumference (OFC) 33 cm (10^th^ centile). Czech consensus anthropometric measures were used for this patient [16]. She was born with navel cord hernia and mild dysmorphic facial features. She walked independently with a clumsy gait from 2 years of age. From age 2, she experienced fever convulsions and recurrent respiratory infections. Neuropsychological testing at age 21 months revealed mild psychomotor retardation and speech delay, with a vocabulary of 20 words and articulation problems. Cerebral magnetic resonance imaging (MRI) and electroencephalography (EEG) examinations were normal. At the clinical examination at age 4, her weight was 19.5 kg (90^th^ centile), height 102 cm (25^th^ centile) and she was microcephalic with an OFC of 46 cm (<3^rd^ centile). Dysmorphic facial features included broad nasal bridge with low nasal root, pear shaped tip of the nose and short columella (Fig. 1a), small chin, low hairline, and low-set ears. She had strabismus. Metabolic screening of urine was normal except from a moderately elevated level of 3-hydroxyisovalerate.

Patient 2, a boy, was the only child to a healthy, non-consanguineous Norwegian couple. He was delivered at term by an unplanned caesarean section due to his large size. Birth weight was 4600 g (97^th^ centile), length 56 cm (>97^th^ centile), and OFC was not recorded. Norwegian consensus anthropometric measures were used for this patient [17]. He gained weight adequately, and walked at 16 months. Lack of interest in the surrounding was noticed by the kindergarten staff. He had impaired hearing due to recurrent middle ear infections, which normalized after he received drainage tubes bilaterally at 4 years of age. Cognitive testing at age 7, using Wechsler Intelligence Scale for Children - third edition 1991 (WISC-III), indicated global developmental delay. Neuropsychological testing with an extensive panel of tests performed at age 8.5 years showed an overall score in the lower normal range. He showed deficits in attention and executive functions. Cerebral MRI and EEG examinations were normal. At the clinical examination at age 9, his height was 125.5 cm (3^rd^ centile), weight 25.7 kg (10^th^ centile) and OFC 52 cm (10^th^ centile). He had delayed fine motor skills, and impaired balance and coordination. He received special tutoring at school. He had mild facial dysmorphisms, including mild hypertelorism, high and broad nasal bridge, low-set ears with thick, upper helixes and hypoplastic cruz superior, full lips, retrognathia, crowded teeth and an open mouth appearance (Fig. 1b-d). He also had mild divergent strabismus and hypermetropia. At age 12 he was diagnosed with pervasive developmental disorder not otherwise specified (PDD-NOS), and found to have borderline cognitive functioning. Testing for fragile X syndrome was negative. Metabolic screening of urine was normal.

## Methods

### Cytogenetic analysis

G-banded karyotyping at 550 band resolution was performed on metaphases from peripheral blood. Array Comparative Genome Hybridization (aCGH) analysis was performed using the Human Genome CGH Microarray 44 K or 244A (Agilent Technologies, Santa Clara, CA) according to the manufacturer’s protocol. Samples were sex-matched with Human Genomic DNA (Promega, Madison, WI). aCGH slides were scanned with the Agilent Microarray Scanner, data obtained using Feature Extraction software (v. 6.1.1) and visualized by Agilent Workbench (v. 3.5.14). CNVs were detected by using the ADM-2 algorithm with three neighboring oligos with aberrant intensity ratios of 0.4 as cut off. All genomic positions were based on the February 2009 human reference sequence (GRCh37/hg19) produced by the Genome Reference Consortium.

### Quantitative Real-time PCR (qPCR)

Quantitative Real-time PCR (qPCR) was performed using the SYBR Green Jump-Start Taq ReadyMix PCR kit (Sigma, Saint Louis, MO). Reactions were run on the ABI PRIMS 7900 HT Sequence Detection System (Life Technologies Corporation, Carlsbad, CA) according to the manufacturer’s recommendations. Amplification levels were calculated using the ∆∆Ct method [18]. Primer sequences are supplied in Additional file 1: Table S1.

Fluorescence *in situ* hybridization (FISH) analysis was performed on chromosomal metaphase spreads from peripheral blood of the maternal grandmother of patient 1, using the BAC clone RP11-41806 (chr2:111631068-111793024 bp) as probe. An Olympus BX 61 fluorescence microscope (Olympus optical company, Tokyo, Japan) with a 1300D CCD camera (Vds Vosskühler, Osnabrück, Germany) was used for image acquisition. Image analysis was performed using the LUCIA-KARYO/FISH software (Laboratory Imaging, Prague, Czech Republic).

### Genomic rearrangement

**Fig. 2:**
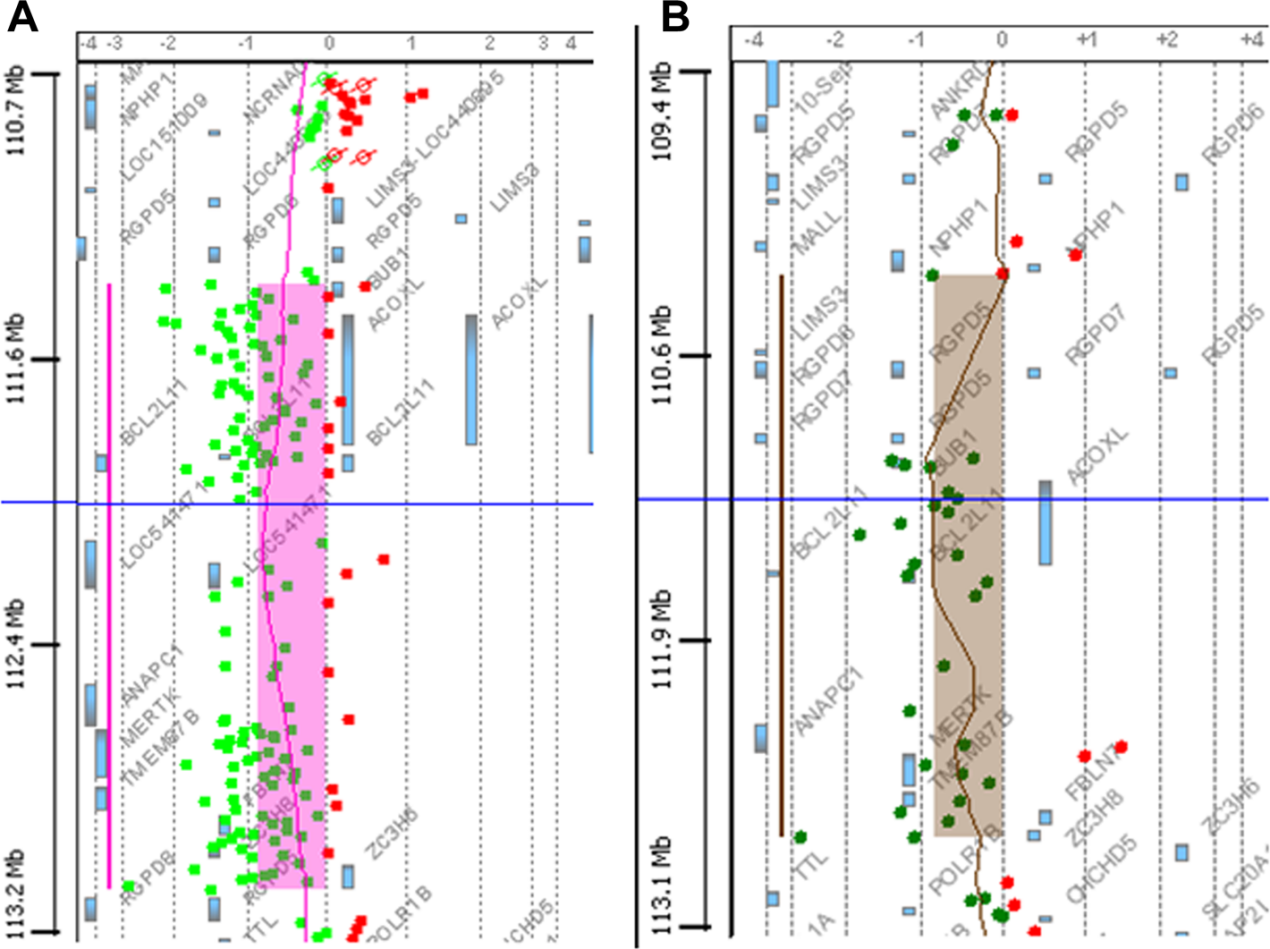
The aCGH results showing the 1.8 Mb and the 2 Mb deletion in 2q13 in patient 1 (**a**) and patient 2 (**b**), respectively

**Fig. 3:**
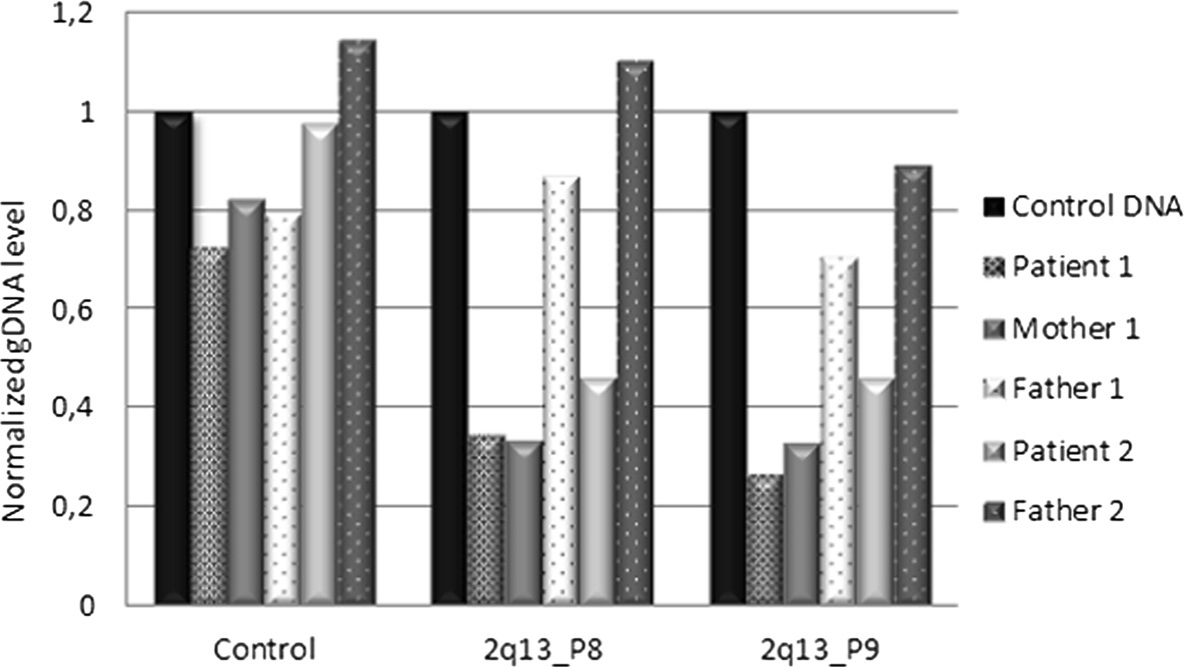
qPCR detected the chromosome 2q13 deletion in patient 1, her mother, and patient 2 using two independent primer pairs, 2q13_P8 and 2q13_P9, as indicated. Normal DNA levels were detected with the control primer set (Control)

Both patients show a normal karyotype by G-banding. Deletions in chromosome 2q13 were detected in both patients by aCGH. A 1.8 Mb deletion in patient 1 (minimal deletion between chr2:111415137-113194067 bp and maximal deletion between chr2:111406838-113210531 bp) and a 2.0-2.2 Mb deletion in patient 2 (minimal deletion between chr2:110980342-113007823 bp and maximal deletion between chr2:110970221-113194067 bp) were identified using a 244 k and 44 k aCGH, respectively (Fig. 2). The presence of the 2q13 deletions was verified in both patients by qPCR analysis (Fig. 3), and was shown to be maternally inherited in patient 1 (Fig. 3). The deletion was also detected in the maternal grandmother of patient 1 by FISH analysis (Additional file 2: Figure S1). The qPCR analysis in the father of patient 2 showed normal results (Fig. 3), but DNA from his mother was unavailable. Further qPCR analysis in patient 2 refined the proximal deletion break point to maximally extend to chr2:110974124 bp, thus not including *NPHP1*, the telomeric deletion break point to maximally extend to chr2:113217195 bp, thus not including *TLL*, and chr2:111400720-113097748 bp was confirmed deleted (Additional file 1: Table S1 and Additional file 3: Figure S2). The overlap of about 1.7 Mb between the minimal deletions in the patients included nine protein-coding genes *BUB1*, *ACOX1*, *BCL2L11*, *ANAPC1*, *MERTK*, *TMEM87B*, *FBLN7*, *ZC3H8* and *ZC3H6*. In addition, the minimal deletion in patient 1 includes *RGPD8* at the telomeric border and the minimal deletion in patient 2 included *RGPD5, RGPD6, LIMS3* at the proximal border of the 2q13 deletion. No additional pathological CNVs were detected.

## Discussion

The cytogenetic band 2q13 is enriched in clusters of segmental duplications (SDs), which may facilitate NAHR resulting in deletions and duplications with variable size and borders in the region chr2:110-114 Mb. These deletions are associated with a developmental syndrome, manifesting with heterogeneous phenotype and incomplete penetrance, which is referred to as 2q13 deletion syndrome. The high prevalence of the 2q13 deletion in patients ascertained for intellectual disability or developmental delay (12 out of 15767) compared to controls (1 out of 8329) reported by Cooper et al. indicates that this deletion is not a benign variant [9], but more research is needed to establish its pathogenicity.

**Fig. 4:**
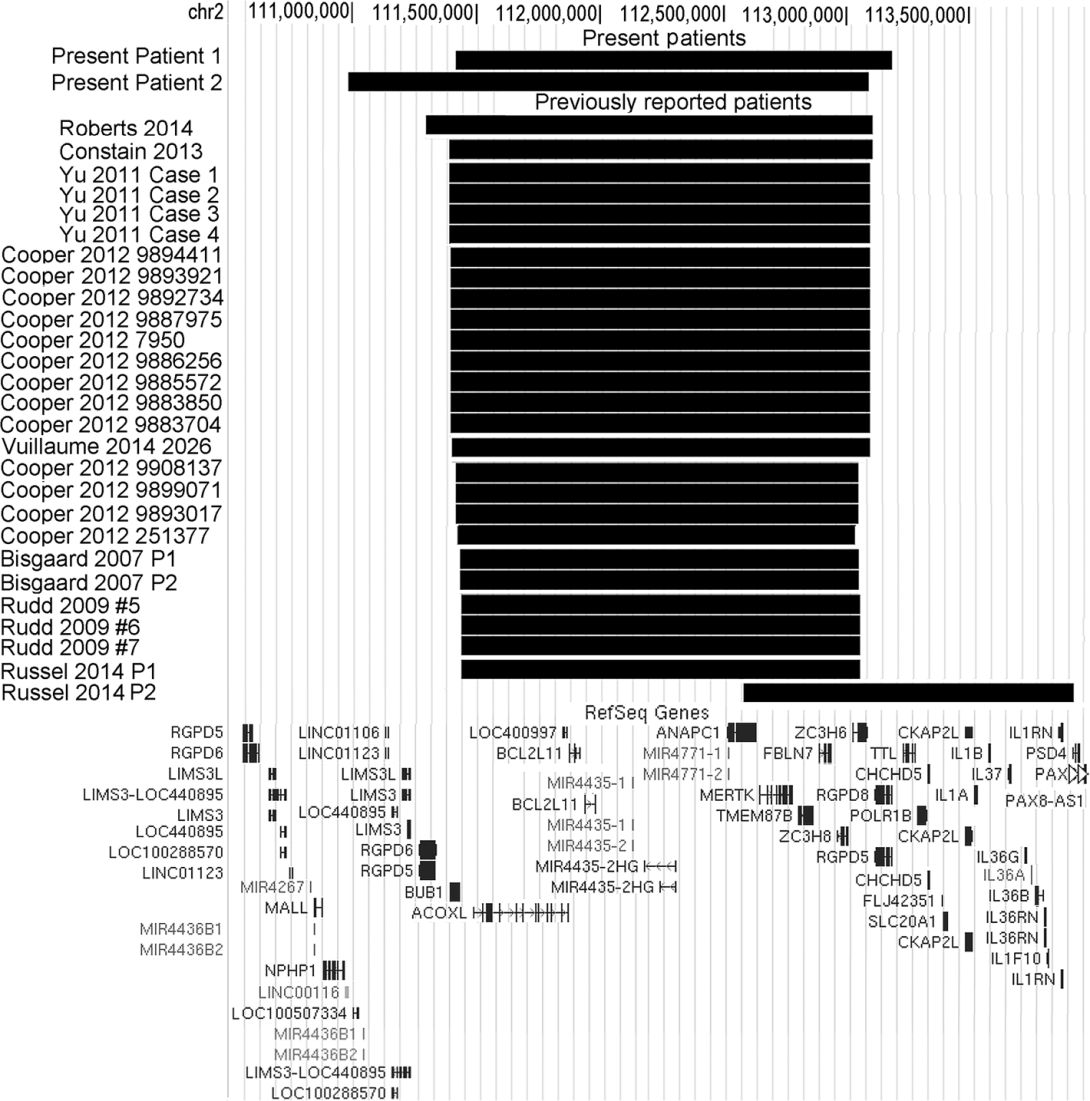
A schematic comparison of the chromosome 2q13 deletions identified in patient 1 and patient 2 reported in the present study and in the 27 previously published patients. The telomeric border of the deletion in patient 2 was refined by qPCR (Additional file 3: Figure S2)

We describe two unrelated patients carrying deletions in 2q13 of 1.8 Mb (patient 1) and 2.0 Mb (patient 2). Patient 1 showed developmental delay, microcephaly and mild dysmorphic features, and patient 2 had ASD, borderline cognitive abilities, deficits of attention and executive functions and mild dysmorphic features. Including the present report, a total of 29 patients with a 2q13 deletion have been described in the literature [8–15] (Fig. 4, Additional file 4: Table S2). It is challenging to compare the clinical features of these patients because most were reported in studies presenting cohorts of patients with limited phenotypic information available for the individual patients. However, 2q13 deletion patients have been described with a developmental syndrome presenting with a combination of the following features (the number of patients reported as having the feature is written in parenthesis): developmental delay/intellectual disability (13), dysmorphic features (13), congenital heart defects (7), hypotonia (7), seizures (5), ASDs (4), macrocephaly (4), microcephaly (3), microphallus (3), and ADHD/ADD (3) (Additional file 4: Table S2). One patient had schizophrenia without ID, ASD or congenital malformations [10]. The 2q13 deletion is also characterized by incomplete penetrance, since unaffected carriers, mostly parents of affected children, have been identified.

Of the 29 patients so far described, two had a verified *de novo* 2q13 deletion (patients 9887950 and 9893017 from [9]). In 11 the deletion was inherited from a healthy parent, and the inheritance was unknown in 14 patients. In the two remaining cases (cases 3 and 4 from [13]), the 2q13 deletion was inherited from parents reported to have a history of developmental delay. However, these parents were not included in the total 29 patients with 2q13 deletions due to the limited information available. In the family of patient 1 in the present report, the 2q13 deletion was identified in two generations of unaffected female carriers.

In the first two described patients carrying the 2q13 deletion and presenting a pathological phenotype, the imbalance was inherited from their healthy mother and therefore concluded to be a benign familial variant [8]. The hypothesis that the 2q13 deletion was pathogenic, but manifesting with high phenotypic variability and incomplete penetrance, was suggested after the detection of the imbalance in three additional patients [11]. Those were part of a cohort of 2419 patients ascertained for developmental delay, ASDs or birth defects and investigated by aCGH analysis. Cooper et al. identified 12 patients with 2q13 deletions among 15767 patients (overall 73 % of cases suffer from ID/DD and/or ASD), and one 2q13 deletion among 8329 controls, supporting the pathogenicity of the 2q13 deletion [9].

There could be several explanations for the variable expressivity and reduced penetrance observed for the 2q13 deletion, such as differences in gene content due to variable deletion break points. It could also be due to differences in genetic background [19], or a recessive allele unmasked by the deletion [20]. According to the “two hit” model, the 2q13 deletion could be a risk factor representing the “first hit”, while the “second hit” could be an additional genomic imbalance, a single mutation in a gene functionally related to a gene in the deletion, or an environmental factor influencing the phenotype [21]. In support of the “two hit” model, three of the 29 patients with a 2q13 deletion patients were reported to carry an additional genomic imbalance (case 1 and 3 from [13]; patient #7 from [11]). In the attempt to detect an unmasked recessive allele, *FBLN7* and *TMEM87B* were sequenced in two 2q13 deletion patients presenting with cardiac defects, but mutations were not found in the remaining alleles [12]. Thus the heterogeneous presentation and the incomplete penetrance of the 2q13 deletion syndrome remain to be elucidated.

Russell et al. depleted the expression of six genes within the 2q13 region, *FBLN7*, *ANAPC1*, *TMEM87B, MERTK, ZC3H8* and *ZC3H6*, in zebrafish in search for the genes responsible for cardiac defects and craniofacial abnormalities associated with the deletion syndrome [12]. Cardiac hypoplasia were seen in animals depleted of *TMEM87B* or *FBLN7*, in addition to craniofacial abnormalities in the latter, while no such abnormalities were monitored in the zebrafish depleted of the four remaining genes [12]. This suggests that heterozygous loss of *FBLN7* and *TMEM87B* contributes to cardiac defects and craniofacial abnormalities associated with 2q13 deletion syndrome. In addition, one or both of these genes could be responsible for additional phenotypes that are not easily monitored in zebrafish, such as developmental delay, or other genes within the region could contribute to some of the remaining phenotypes. For example *BCL2L11,* encoding a Bcl2-like antiapoptotic protein with a role in neuronal apoptosis, has been found down regulated in individuals with ASDs [22–24], a diagnosis given to three of the 29 patients with 2q13 deletion.

## Conclusions

Due to limited number of patients reported with smaller, atypical deletions, and limited functional information about several of the genes included in the deletion, it is challenging to establish genotype-phenotype correlations. However, *FBLN7* and *TMEM87B* likely contribute to the cardiac defects and craniofacial dysmorphisms [12]. Another gene of interest is *BCL2L11*, suggested to play a role in the development of ASDs [22–24]. Despite several 2q13 deletion patients described and functional studies performed, the understanding of the genotype-phenotype correlations and of the mechanisms underlying the heterogeneous presentation and incomplete penetrance in this syndrome is limited. Further functional studies of the 2q13 genes, additional reports of patients with smaller, atypical deletions as well as the identification of mutations in single genes are needed to establish the pathogenicity of the deletion and improve the understanding of the 2q13 deletion syndrome.

## Consent

Written informed consent was obtained from the patient for publication of this Case report and any accompanying images. A copy of the written consent is available for review by the Editor-in-Chief of this journal.

## Additional files

Additional file 1: Table S1. Primer pairs used in qPCR experiments. Additional file 2: Figure S1. FISH on metaphase chromosomes from the maternal grandmother of patient 1:RP11-41806 (chr2: chr2:111631068-111793024 bp, green) gave one signal, documenting the deletion. Control for chromosome 2: 2p subtelomere probe (VIJyRM2052, red). Additional file 3: Figure S2. 2q13 deletion break points in patient 2 were refined by qPCR analysis using seven primer pairs at the proximal break point (2q13_P1-P7) and at the terminal break point (2q13_P10-P16). gDNA levels in the patient was normalized to control DNA set to 1. At the proximal break point, genomic regions amplified by 2q13_P1-P3 gave normal results, whereas primers 2q13_P4-P7 showed that this region was deleted. At the telomeric break point, the genomic regions amplified by 2q13_P10-P14 were deleted, whereas genomic regions amplified by 2q13_P15 and 2q13_P16 gave normal results (primer sequences and genomic regions of amplicons are given in Additional file 1: Table S1). Additional file 4: Table S4. Summary of CNVs and clinical features in the 29 patients with 2q13 deletion.
