# Supplementary figures and images for: A recurrent deletion on chromosome 2q13 is associated with developmental delay and mild facial dysmorphisms
Source: Mol Cytogenet. 2015 Jul 31;8:57. doi: 10.1186/s13039-015-0157-0 (PMC4521466; doi:10.1186/s13039-015-0157-0)

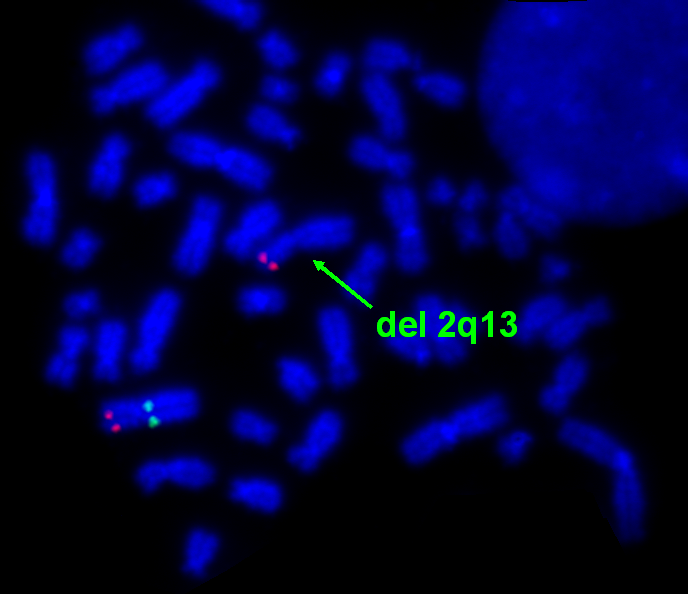

Supplement: Additional file 2: Figure S1. — FISH on metaphase chromosomes from the maternal grandmother of patient 1:RP11-41806 (chr2: chr2:111631068-111793024 bp, green) gave one signal, documenting the deletion. Control for chromosome 2: 2p subtelomere probe (VIJyRM2052, red). [file 13039_2015_157_MOESM2_ESM.tif]

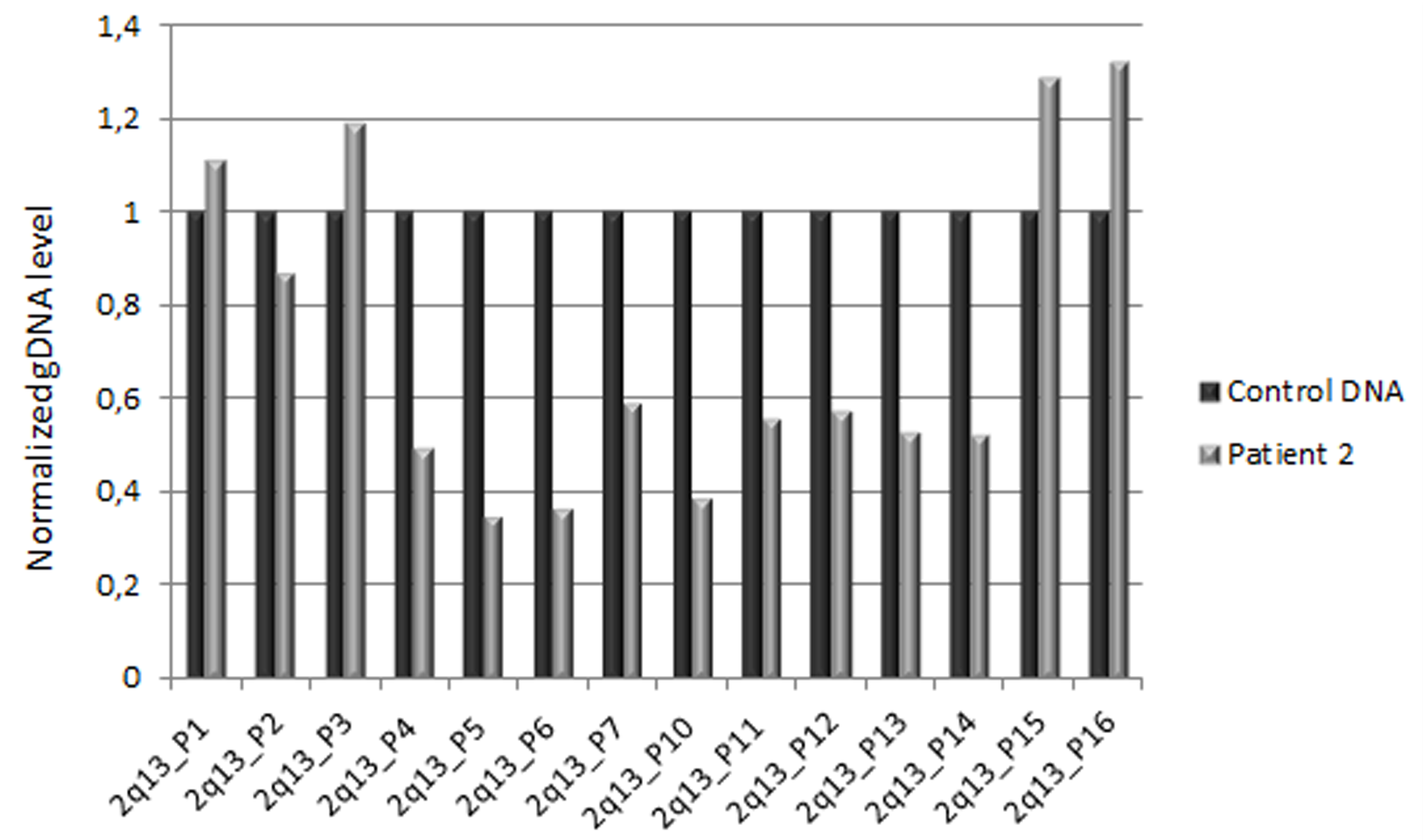

Supplement: Additional file 3: Figure S2. — 2q13 deletion break points in patient 2 were refined by qPCR analysis using seven primer pairs at the proximal break point (2q13_P1-P7) and at the terminal break point (2q13_P10-P16). gDNA levels in the patient was normalized to control DNA set to 1. At the proximal break point, genomic regions amplified by 2q13_P1-P3 gave normal results, whereas primers 2q13_P4-P7 showed that this region was deleted. At the telomeric break point, the genomic regions amplified by 2q13_P10-P14 were deleted, whereas genomic regions amplified by 2q13_P15 and 2q13_P16 gave normal results (primer sequences and genomic regions of amplicons are given in Additional file 1: Table S1). [file 13039_2015_157_MOESM3_ESM.tif]
